# Immune-Related Long Non-coding RNA Constructs a Prognostic Signature of Ovarian Cancer

**DOI:** 10.1186/s12575-021-00161-9

**Published:** 2021-12-15

**Authors:** Xiaoyu Sun, Shan Li, Xuemei Lv, Yuanyuan Yan, Minjie Wei, Miao He, Xiaobin Wang

**Affiliations:** 1 Department of Pharmacology, School of Pharmacy, China Medical University, Shenyang, Liaoning Province China; 2 Liaoning Key Laboratory of Molecular Targeted Anti-tumor Drug Development and Evaluation, Liaoning Cancer Immune Peptide Drug Engineering Technology Research Center, Key Laboratory of Precision Diagnosis and Treatment of Gastrointestinal Tumors (China Medical University), Ministry of Education, Shenyang, Liaoning Province China; 3 Department of Breast Surgery, The First Affiliated Hospital of China Medical University, Shenyang, Liaoning Province China; 4 Shenyang Kangwei Medical Laboratory Analysis Co. LTD, Shenyang, Liaoning Province China; 5 Center of Reproductive Medicine, Shengjing Hospital of China Medical University, Shenyang, China

**Keywords:** Ovarian cancer, Long non-coding RNA, Immune infiltration, Signature, Chemo-sensitivity

## Abstract

**Background:**

Since ovarian cancer leads to the poor prognosis in women all over the world, we aim to construct an immune-related lncRNAs signature to improve the survival of ovarian cancer patients.

**Methods:**

Normal and cancer patient samples and corresponding clinical data of ovarian were obtained from The Genotype-Tissue Expression (GTEx) portal and The Cancer Genome Atlas (TCGA) database. The predictive signature was constructed by the lasso penalty Cox proportional hazard regression model. The division of different risk groups was accounting for the optimal critical value of the time-dependent Receiver Operating Characteristic (ROC) curve. Finally, we validated and evaluated the application of this prognostic signature based on the clinical factors, chemo-sensitivity and immune status of different risk groups.

**Results:**

The signature was established from 145 DEirlncRNAs and can be shown as an independent prognostic risk factor with accurate prediction on overall survival in ovarian cancer patients. Further analysis on the application of the prognostic signature showed that patients with low-risk had a better sensitivity to chemotherapy and a higher immunogenicity.

**Conclusion:**

We constructed and verified an effective signature based on DEirlncRNA pairs, which could predict the prognosis, drug sensitivity and immune status of ovarian cancer patients and promote the prognostic estimation and individualized treatment.

**Supplementary Information:**

The online version contains supplementary material available at 10.1186/s12575-021-00161-9.

## Introduction

Ovarian cancer (OC) is one of the most common cancer in gynecological tumors which has the highest mortality rate [1]. Most of the OC patients were usually diagnosed at advanced stage with a poor prognosis since there is no symptom at early stage [2]. Although the development of cancer treatment was quickly in recent years, different subtypes of OC based on biological and molecular characteristics lead to a low rate of 5-year survival at 47% [2, 3]. At present, the first choice for the treatment of OC is still surgery and systemic therapy. The limitation of treatment response and the cancer recurrence caused by drug resistance remain us that it is necessary to screen effective therapy strategies and prognostic biomarkers for OC [4]. In addition to poly (adenosine diphosphate-ribose) polymerase (PARP) inhibitors, immunotherapy has shown the potential in targeted therapy of OC [5, 6]. The important role of the immune system in cancer was improved by the application of immune checkpoint inhibitors (ICIs) in various cancers such as liver cancer, melanoma and prostate cancer [7–10]. There is evidence that the objective response rate (ORR) of the combined use of programmed death ligand 1 (PD-L1) inhibitors and PARP inhibitors is only about 19% [11]. Thus, risk stratification based on immune factors is necessary for predicting the therapeutic sensitivity and prognosis in OC.

Long non-coding RNAs (lncRNAs) are non-translational RNAs with transcripts of more than 200 nucleotides, which have been shown to regulate a series of biological processes and play an indispensable role in cancer development [6, 12]. LncRNAs are differentially expressed and act as a potential biomarker in OC, which can promote the occurrence, metastasis, drug resistance, and lead to poor prognosis [13, 14]. LncRNAs can act as regulators in the immune system in spite of the non-function on coding immune-related proteins [15]. LncRNASNHG12 and HOTTIP were found to promote immune escape of ovarian cancer cells [16, 17]. The effect of immune-related lncRNAs (irlncRNAs) has attracted extensive attention in the prognosis of liver cancer, breast cancer, and other cancers [18–21]. Therefore, irlncRNAs, a combined model, shows great promising prognostic and predictive value in the diagnosis, evaluation, and management of cancer [22].

The prognostic value of irlncRNAs in OC has not been systematically determined yet. This study applied a novel algorithm in constructing an irlncRNAs signature independent of the specific expression of the certain lncRNA. Subsequently, we further validated the clinical value of the prognostic signature and confirmed that this signature can be used as a predictor of chemo-sensitivity and immuno-sensitivity of OC.

## Materials and Methods

### Data Collection

The RNA-seq data and clinical information of OC samples were collected and downloaded through The Cancer Genome Atlas database (TCGA, https://tcga-data.nci.nih.gov/tcga/). Using The Genotype-Tissue Expression database (GTEx, https://gtexportal.org/home/) portal, the expression of genes analyzed in normal ovarian samples was collected. A total of 88 normal samples and 379 ovarian cancer samples were collected. Batch correction was performed using the R package *sva* (R software 4.0.4). To differentiate the mRNAs and lncRNAs, we downloaded the GTF files from Ensembl (http://asia.ensembl.org) as annotation for subsequent analysis. Besides, we download a recognized immune-related genes (ir-genes) list in the Immunology Database and Analysis Portal database (ImmPort, http://www.immport.org).

### Extraction and Differential Expression Analysis of irlncRNAs

Through the co-expression analysis, the correlation analysis between ir-genes and lncRNAs was performed. IrlncRNA was defined as immune gene whose correlation coefficient was more than 0.4 and *p* < 0.001. Different expression among irlncRNAs was analyzed by R package *limma* with the log2 fold change (log_2_FC) > 2 and false discovery rate (FDR) < 0.5 were considered as DEirlncRNAs.

### Establishment of DEirlncRNA Pairs

In order to construct a 0–1 matrix, we compared each DEirlncRNA in a single cycle. If the expression of lncRNA *A* was higher than that of lncRNA *B*, it was affirmed as 1; otherwise, it was affirmed as 0. If the gene had a consistent expression in all patients, it could not make sense to prognosis. When the 0 or 1 expression quantity of lncRNA-pair ranged from 20 to 80%, it was considered as a valid match.

### Establishment of Risk Assessment Signature

Univariate analysis of DEirlncRNAs based on survival was performed, and then optimized by Lasso regression analysis and cross validation via *glmnet* package of R. Finally, a risk assessment signature related to prognosis was constructed. The 1-, 3-, and 5-year receiver operating characteristic (ROC) curves of the signature were performed. After that, we calculated the risk score through the following formula: β1*expr 1 + β2*expr 2 + …… + βn*expr n (βn refer to regression coefficient of DEirlncRNA pairs and expr n refer to expression of the matched DEirlncRNA pairs). By evaluating the AUC of the above three ROC curves, we determined that the AUC value of the 5-year ROC curve was the maximum. According to the cut-off value of the 5-year ROC curve, ovarian cancer patients were divided into high-risk group and low-risk group, respectively.

### Verification of Risk Assessment Signature

To verify the predictive value of this signature, we performed the ROC curve analysis, Kaplan-Meier log-rank test, independent prognostic analysis and multivariate Cox regression analyses to compare survival between the high-risk and low-risk groups. We utilized three R packages including *survival, survivalROC* and *survminer*.

### Clinical Evaluation of Risk Assessment Signatures

After validation, differences between riskScore and clinicopathologic characteristics were assessed by the Wilcoxon rank-sum test, and the results were presented as box diagram and statistical significance was labeled as follows: *p* < 0.001 = ∗∗∗, *p* < 0.01 = ∗∗, and *p* < 0.05 = ∗. Next, we explored the significance of risk signatures in clinical chemo-sensitivity. We included and processed the half inhibitory centration (IC_50_) of chemotherapy drugs recommended in the The American Joint Committee on Cancer (AJCC) guidelines for ovarian cancer therapy. Drugs we analyzed were platinum, paclitaxel, PARP inhibitors, etc. The Wilcoxon rank-sum test was used to compare IC_50_ between high-risk and low- risk groups and the results were also shown on the box diagram. The R packages utilized included: *limma, ggpubr, pRRophetic*, and *ggplot2*.

### Tumor Immunology Analysis

In order to figure out the relationship between this signature and tumor immune microenvironment, we analyzed the expression of immune cells between the high and low-risk groups. Results with *p* < 0.05 were shown on box chart and box diagrams. This procedure was performed using R package *ggplot2*.

## Results

### IrlncRNAs in Ovarian Cancer

**Fig. 1:**
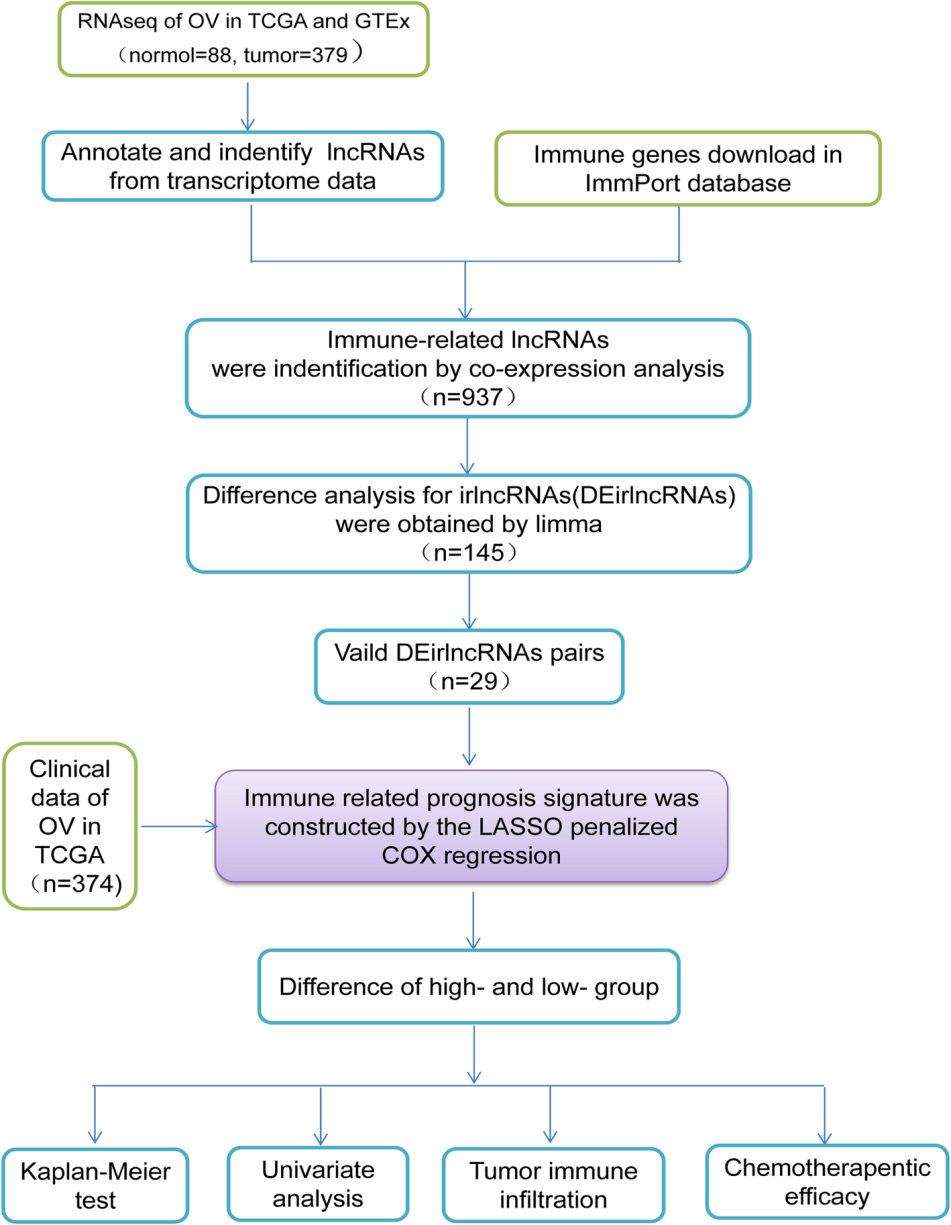
Flow Diagram of this study

Figure 1 shows the flow diagram of this study. Transcriptome data of OC downloaded from TCGA and GTEx included 88 normal samples and 379 cancer samples. Two thousand four hundred eighty-three immune-related genes were obtained from the ImmPort database. The co-expression analysis of immune genes and lncRNAs obtained 616 immune-related lncRNAs was shown in Table S1 (*p* < 0.001).

### Establishment of Risk Assessment Signature by Differentially Expressed irlncRNA (DEirlncRNA) Pairs

**Fig. 2:**
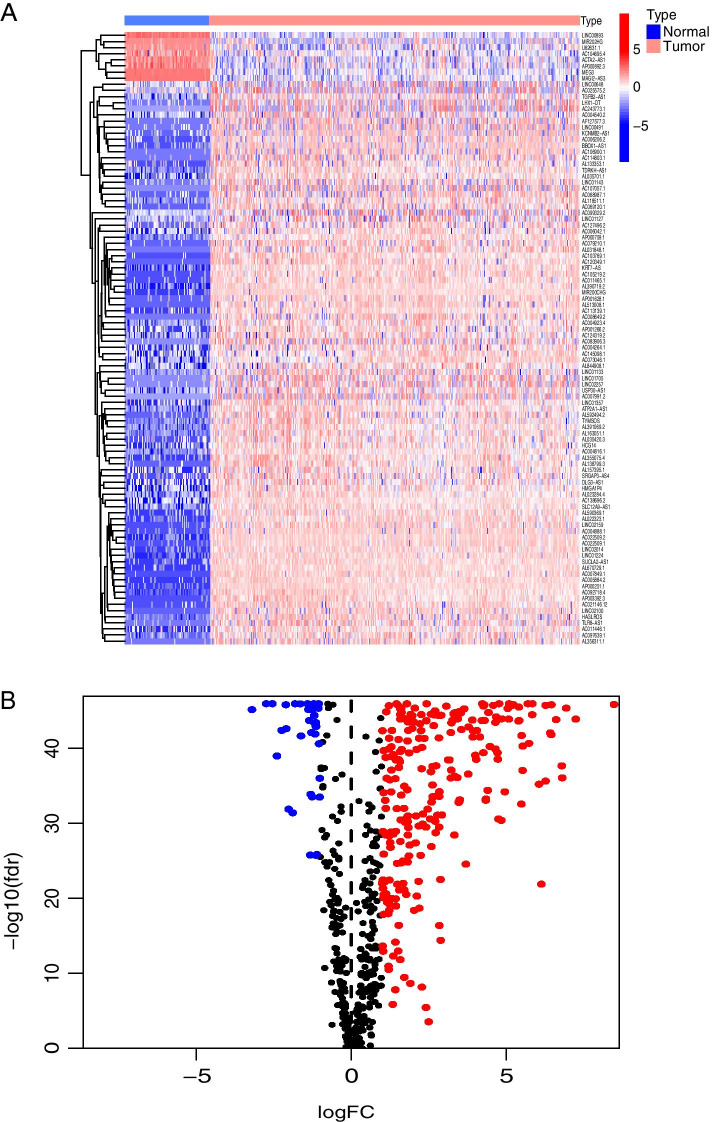
Extraction and differential expression analysis of irlncRNAs. Extraction of DEirlncRNAs using TCGA and GTEx datasets. Heat map (**A**) and volcano plot (**B**) of immune-related Long non-coding RNAs were presented

**Fig. 3:**
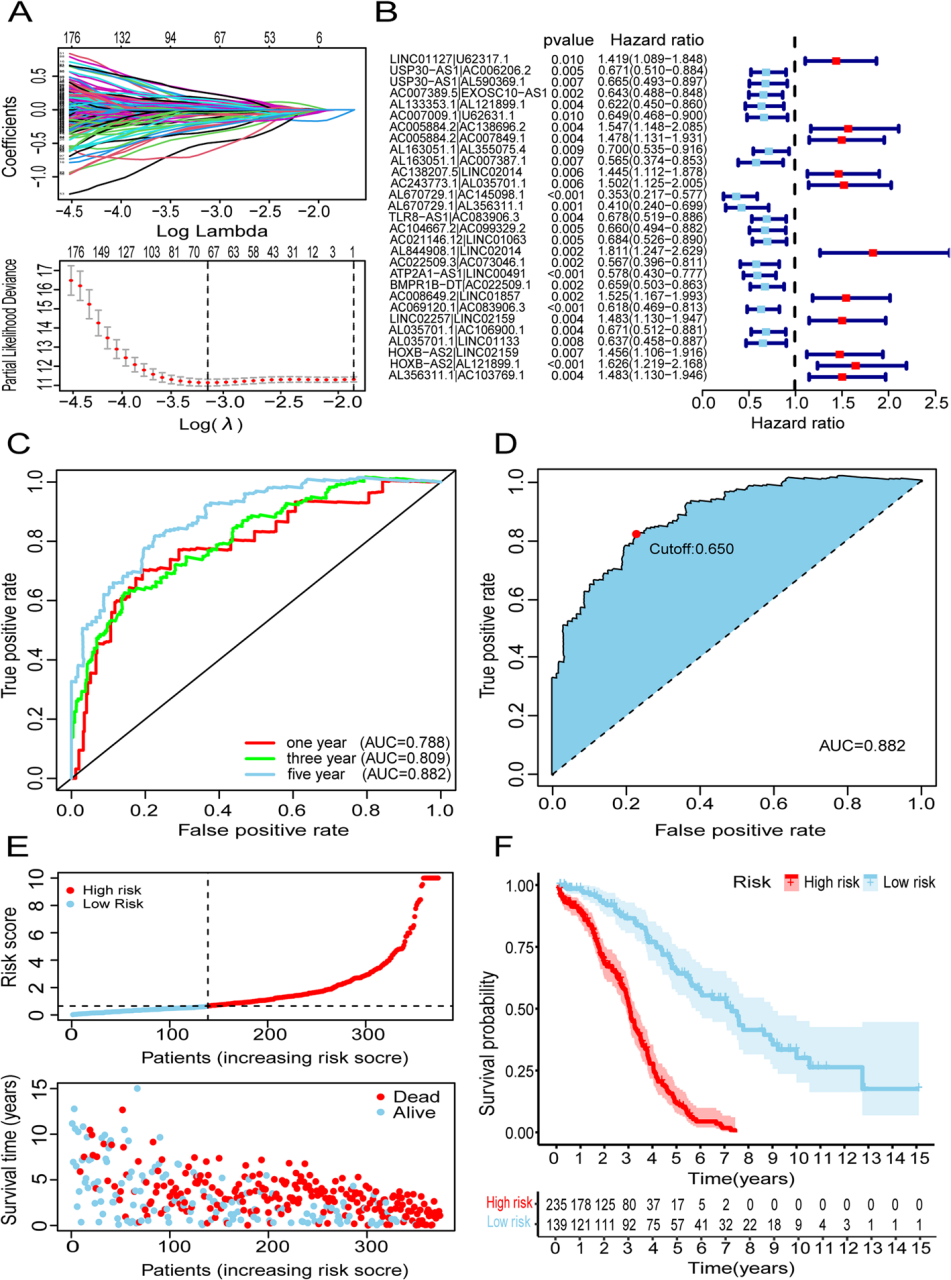
Establishment of risk assessment signature by DEirlncRNA pairs. **A** LASSO coefficient profiles of the 29 DEirlncRNA pairs. **B** Forest plot of 29 DEirlncRNA pairs selected by univariate Cox regression analysis. **C** Plot a curve of every AUC value generated by 1-, 3-, and 5-year ROCs of 29 DEirlncRNA pairs signature. **D** The maximum AUC value was that of the 5-year ROC curve. **E** Distribution of risk score, survival status of each patients of risk assessment signature in high-risk and low-risk groups were presented. **F** Kaplan–Meier curve of the high-risk and low-risk groups were presented

We obtained 145 DEirlncRNAs in the subsequent differential analysis. One hundred thirty-seven of the one hundred forty-five DEirlncRNAs were up-regulated and 8 of them were down-regulated (Fig. 2A and B). Based on the 145 DEirlncRNAs, a 0–1 matrix was constructed through a single cycle comparation, and 8613 valid pairs of DEirlncRNA were obtained. Single factor test and Lasso regression analysis were used to optimize and screen the DEirlncRNA pairs (Fig. 3A). So as to avoid over-fitting and improve the accuracy of the signature, we used the cross-validation and finally gained 29 DEirlncRNA pairs (Fig. 3B).

A total number of 374 available ovarian cancer patients’ samples from the TCGA database were used to calculate the risk scores, and the 29 DEirlncRNA pairs were used to calculate the areas under curve (AUCs) for each ROC curve. In order to confirm the optimality, we performed the 1-, 3-, and 5-year ROC curves and found the maximum area under the curves belonged to the 5-year ROC curve, which was selected to distinguish the high and low-risk groups of the signature (Fig. 3C and D). Finally, we obtained 235 cases in the high-risk group and 139 cases in the low-risk group (Fig. 3E). Further, we verified the signature based on prognosis. The risk coefficient was positively related to the mortality, and the Kaplan-Meier analysis also confirmed that the low-risk group patients had a longer survival.

### Validation of the Clinical-Based Risk Assessment Signatures

**Fig. 4:**
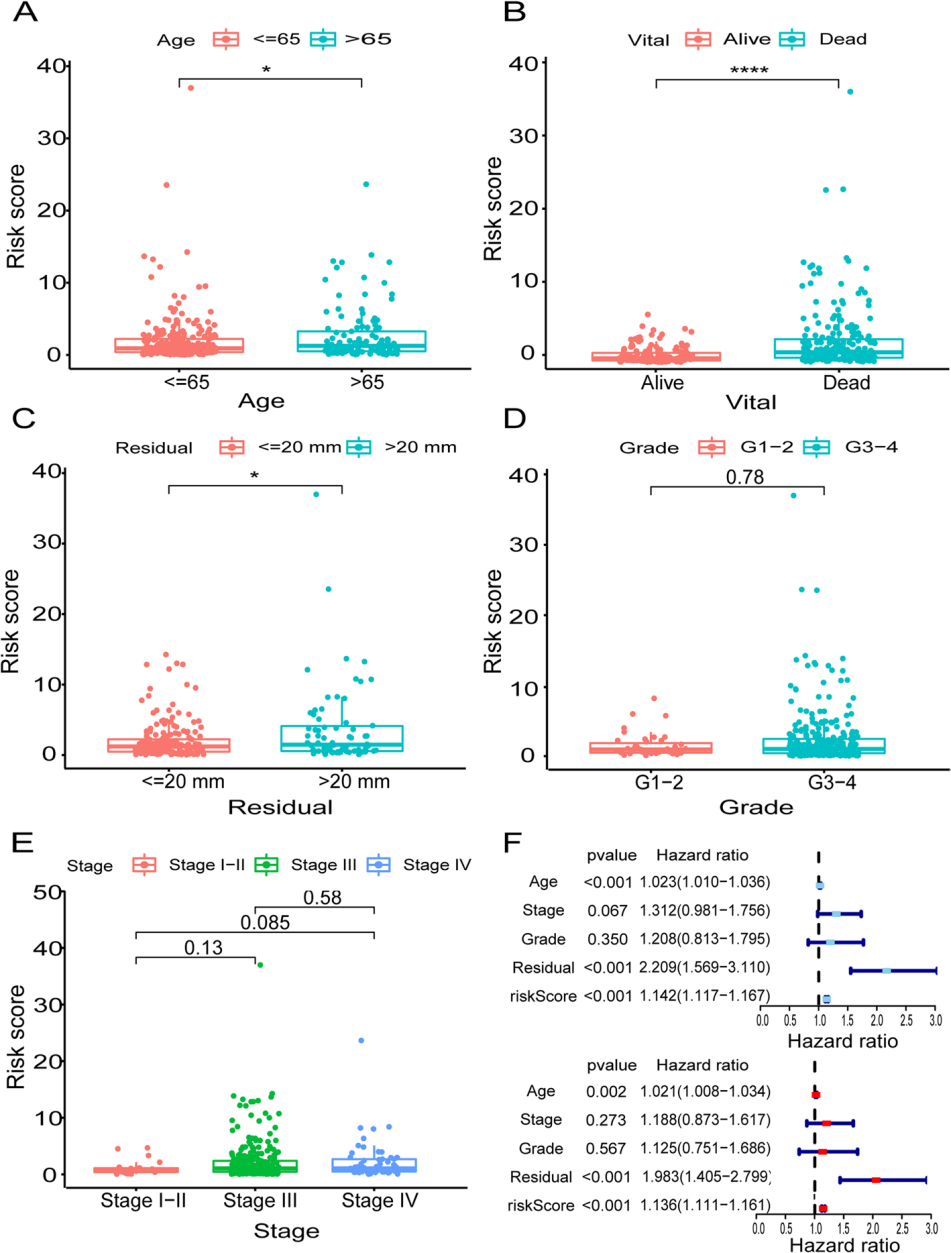
Clinicopathological factors assessment by risk assessment signature. The box-plot showed that there were statistical difference expressions of the risk assessment signature in age (**A**), vital (**B**) and residual (**C**). There were no significant correlations with, respectively, grade (**D**) and stage (**E**). A univariate Cox regression analysis and multivariate Cox regression demonstrated that riskScore presented as an independent prognostic predictor

In order to figure out the relationship between this risk signature and different clinicopathological factors, we performed the Wilcoxon rank-sum test and the results showed the risk was significantly related to age (*p* < 0.05), survival status (*p* < 0.0001) and residual tumor lesions (*p* < 0.05, Fig. 4A-C). There was no significant correlation with OC grade and stage (Fig. 4D and E). However, there was some difference between the stage I-II and stage IV (*p* = 0.085). Next, we verified the risk signature as an independent prognostic factor for OC through the univariate and multivariate Cox regression analyses. The results indicated that age (*p* < 0.001, HR = 1.023, 95%CI [1.010–1.036]), residual tumor lesions (*p* < 0.001, HR = 2.209, 95%CI [1.596–3.110]) and riskScore (*p* < 0.001, HR = 1.142, 95% CI [1.117–1.167]) was related to overall survival in univariate Cox regression analysis (Fig. 4F). In multivariate Cox regression analysis, riskScore could be used as an independent predictor of prognosis (Fig. 4F, *p* < 0.001, HR = 1.136, 95% CI [1.111–1.161]).

### Application of Risk Assessment Signature in Chemo-sensitivity

**Fig. 5:**
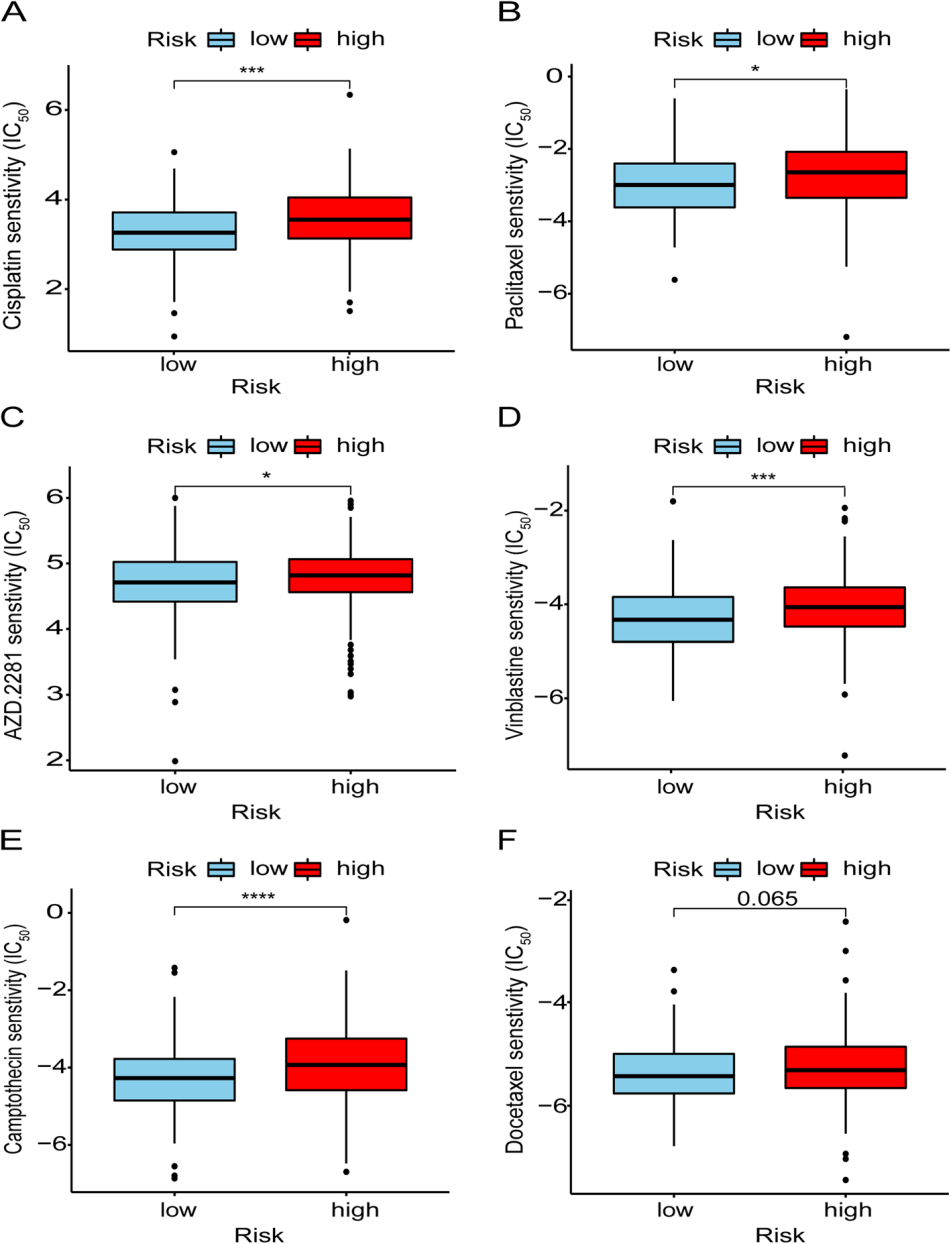
Chemo-sensitivity assessment by risk assessment signature. The box-plot showed that there were difference expressions of the risk assessment signature in cisplatin (**A**), paclitaxel (**B**), AZD.2281 (**C**), vinblastine (**D**), camptothecin (**E**) and docetaxel (**F**)

Furthermore, we also analyzed the efficacy of common chemotherapy drugs for OC by the IC_50_. From the box diagram, we could find that the low-risk group had higher sensitivity to chemotherapy. Among them, platinum (*p* < 0.001) and paclitaxel (*p* < 0.05), the most conventional chemotherapy drugs for ovarian cancer, had statistical differences (Fig. 5A and B). PARP inhibitors, a novel chemotherapy agent for ovarian cancer, also showed lower IC_50_ values in the low-risk group (Fig. 5C, *p* < 0.05). Besides, vinblastine (*p* < 0.001) and camptothecin (*p* < 0.0001) also differed in drug sensitivity among patients at different risk (Fig. 5D and E). Docetaxel, although not statistically significant, did show different drug sensitivities in different risk groups (Fig. 5F, *p* = 0.065). This suggests the possibility of the label as a predictor of chemotherapeutic drug sensitivity.

### Correlation of Tumor Immunotherapy and Risk Assessment Signature

**Fig. 6:**
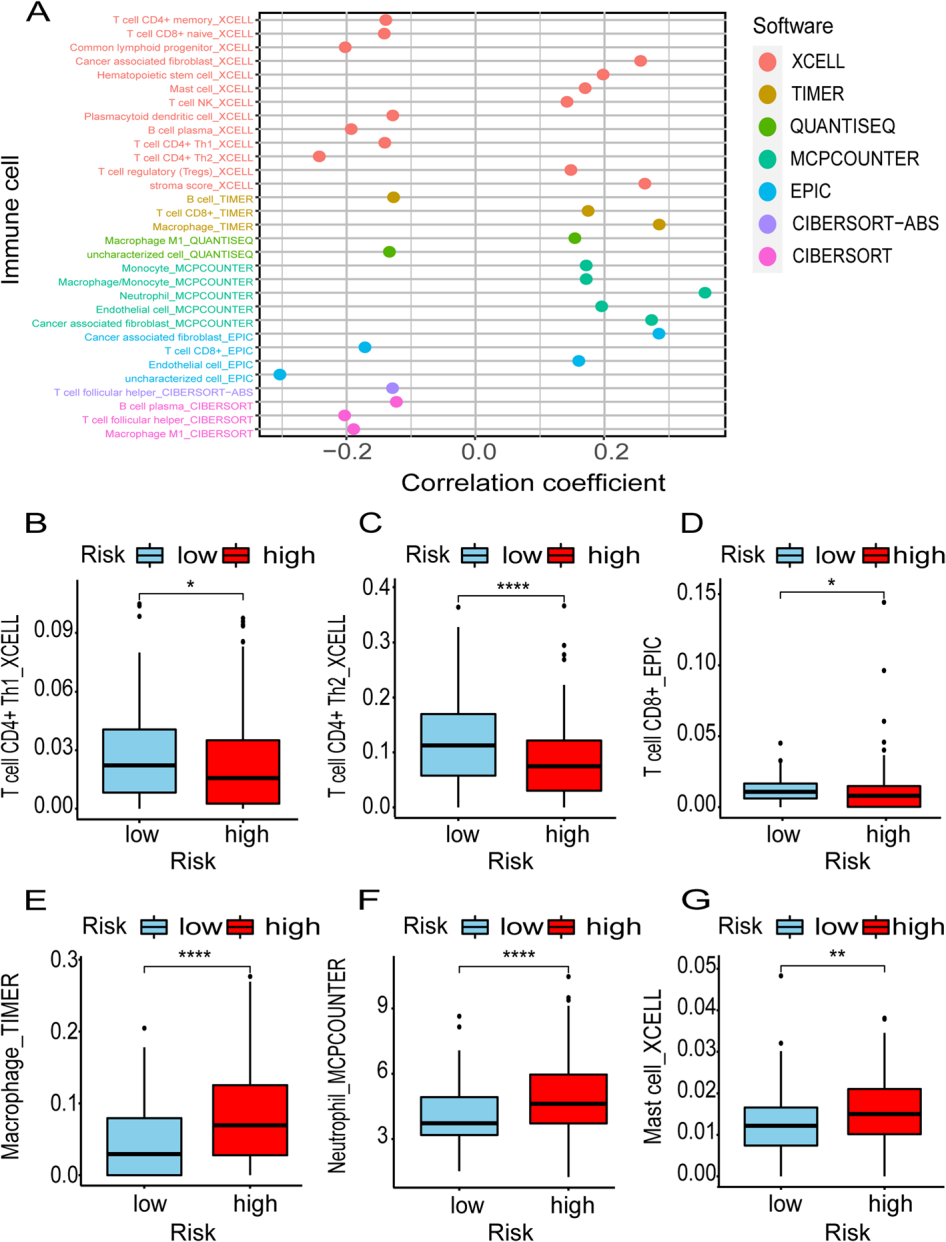
Tumor immunotherapy assessment by risk assessment signature. Risk signature was related to tumor-infiltrating immune cells (**A**). The box-plot showed that the low-risk group was positively correlated with specific immune cells, such as B cells and T cells (**B-D**), while was negatively correlated with non-specific immune cells, such as neutrophils, macrophages and mast cells (**E-G**)

Clinical application of immunotherapy has attracted widespread attention in ovarian cancer. We investigated whether this risk signature was correlated with tumor-infiltrating immune cells (Fig. 6A). The figure showed differences in the expression of immune cells in the high and low-risk groups. We found that the low-risk group had positive correlation with specific immune cells, such as B cells and T cells (Fig. 6B-D), while had negative correlation with non-specific immune cells, such as neutrophils, macrophages and mast cells (Fig. 6E-G). This suggests the possibility of the risk signature on immunotherapy in ovarian cancer.

## Discussion

With the deep researches in cancer, immunotherapy has become one of the routine methods for cancer treatment [23]. To date, ICIs targeting three different molecules has been approved for human use by the US Food and Drug Administration (FDA), namely antibodies against PD-1/PD-L1 and CTLA-4 [24]. Currently, PD-1/PD-L1 antibodies are used in a wide variety of cancers, however, the effect of immunotherapy were still not satisfactory in OC [25]. Recently, lncRNAs have been proved to play a vital role in regulating the immune system by affecting tumor microenvironment, epithelial-mesenchymal transformation, activation and differentiation of T cells and B cells [15, 26–29]. IrlncRNAs are closely related to immune infiltration and have greater value on predicting prognosis for various types of cancer such as liver cancer, cervical cancer, breast cancer and so on [18, 19, 21]. Cao et al. [30] developed a powerful prognostic signature with 33 irlncRNA pairs in lung adenocarcinoma (LUAD) and validated using the Gene Expression Omnibus (GEO) database, which showed great potential as a prognostic biomarker and a predictor of immunotherapy in LUAD. Coincidentally, the prognostic model of gastric cancer based on 15 irlncRNA pairs offered great promise in predicting the prognosis, immune infiltration status, and chemotherapeutic efficacy in gastric cancer [31]. Thus, it is necessary to construct a prognostic model which can predict the prognosis and immunotherapy response based on immune-related lncRNAs in ovarian cancer.

Liang et al. [32] used weighted gene co-expression network analysis (WGCNA) and found that the expression levels of 4 lncRNAs could be used as independent immune prognostic factors for OC. Liu et al. [33] identified 52 lncRNAs as ovarian cancer-specific immune lncRNA and redefined two different molecular subtypes. Different from the existing researches, we established a new algorithm of irlncRNAs pairs in OC by screening DEirlncRNAs in normal and cancer samples. This signature was based on the relative expression level of each two lncRNAs rather than the specific expression of each lncRNA. In order to ensure the accuracy and effect of the DEirlncRNA pairs, we used single factor test, Lasso regression analysis and cross-validation to optimize our signature. Median value was widely used as the cut-off point to distinguish subgroups in traditional prognostic models. In contrast to the median score, we determined the optimal tipping point by calculating each AUC value for different ROCs to distinguish the different risk groups. In order to confirm the superiority of our prognostic signature with traditional prognostic models, we verified the excellent ability of this signature on classifying patients to different risk groups associated with prognosis. Furthermore, we validated the signature through various clinicopathological factors. Finally, we demonstrated that there was significant relationship between risk classification and chemotherapy sensitivity, which might indicate the potential application of this novel signature on OC treatment.

Immunotherapy has already become a novel treatment strategy for OC [34]. Compared to melanoma, bladder cancer, and breast cancer, the effect of immunotherapy in OC is still modest, most likely due to indolent anticancer immunity [35]. Hamanishi et al. [36] reported the best overall response rate in 20 assessable patients treated with nivolumab was 15%. The combination of nivolumab and ipilimumab in EOC produced a higher remission rate and a longer PFS, but it was limited [37]. Recently, a Phase III clinical trial also showed that OC patients didn’t obtain a significant benefit in immunotherapy [38]. It is necessary to select the population with higher sensitive to immunotherapy. Our results suggested that the distribution of immune-related genes was significantly different in the different risk groups. Patients with low-risk have a higher infiltration level of immune cells including CD4^+^ T cells and CD8^+^ T cells, which might have higher immunogenicity and more suitable for immunotherapy. Consistent with the previous researches, we found that an abundance of CD4^+^ T cells and CD8^+^ T cells could play an important role in immunotherapeutic response [39–41]. It is concerned that due to the immunotherapy as a monotherapy have the unsatisfactory results, accumulated evidence indicates the clinical efficacy of combining appropriate dose of chemotherapies with ICIs [42]. It is necessary to explore the combination of immunotherapy and chemotherapy or PARP inhibitors in OC [43, 44]. Our predictive signature can be used to analyze the sensitivity of chemotherapy and immunotherapy in different risk groups, which may provide a potential possibility for the combination of these two kinds of therapy strategies in OC.

Due to the differences in gene expression levels of different patients, external validation was necessary to the predictive signatures based on the exact gene expression. Our signature was constructed based on the relative expression on lncRNAs pairs, which can avoid the errors caused by the difference of gene expression level to the greatest extent. Nevertheless, there are still some limitations in the study. Firstly, the data set was relatively scarce because the original data we used only included TCGA and GTEx database. Secondly, even we confirmed the signature by various methods, the signature was still lack of the external verification to make it more convince. Finally, we hope to expand our clinical sample size, through further work to improve our model and determine its clinical value.

## Conclusions

In conclusion, we successfully constructed a novel predictive signature on the basis of DEirlncRNA pairs, which could predict the prognosis, chemotherapy sensitivity of OC patients, as well as help to distinguish the patients who can benefit from immunotherapy. We hope that this signature can be applied on predicting the survival of OC patients, also have influence on guiding immunotherapy and chemotherapy management in the future.

## Supplementary Information

**Additional file 1.**

**Additional file 2.**

## Data Availability

All data included in this study are available including The Cancer Genome Atlas (TCGA, https://cancergenome.nih.gov/), The Genotype-Tissue Expression (GTEx https://gtexportal.org/home/) portal and Immunology Database and Analysis Portal (ImmPort, https://immport.org/home).

## Abbreviations

GTEx: The Genotype-Tissue Expression
TCGA: The Cancer Genome Atlas
ROC curve: Receiver Operating Characteristic curve
OC: Ovarian cancer
PARP: Poly (adenosine diphosphate-ribose) polymerase
ICIs: Immune checkpoint inhibitors
ORR: Objective response rate
PD-L1: Programmed death ligand 1
lncRNAs: Long non-coding RNAs
irlncRNAs: Immune-related lncRNAs
DEirlncRNAs: Differentially Expressed irlncRNAs
ImmPort: Immunology Database and Analysis Portal
AUC: Areas under curve
IC_50_: Half inhibitory centration
AJCC: The American Joint Committee on Cancer
FDA: Food and Drug Administration
LUAD: Lung adenocarcinoma
GEO: Gene Expression Omnibus
WGCNA: Weighted gene co-expression network analysis
