# Supplementary material for: Immune-Related Long Non-coding RNA Constructs a Prognostic Signature of Ovarian Cancer
Source: Biol Proced Online. 2021 Dec 15;23:24. doi: 10.1186/s12575-021-00161-9 (PMC8903634; doi:10.1186/s12575-021-00161-9)
Supplement: Supplementary file 2 — Additional file 2. [file 12575_2021_161_MOESM2_ESM.doc]

| **immune** | **cor** | **pvalue** |
| --- | --- | --- |
| B cell plasma_CIBERSORT | -0.122793564 | 0.046235042 |
| B cell plasma_XCELL | -0.192802147 | 0.001647401 |
| B cell_TIMER | -0.127134182 | 0.038992367 |
| Cancer associated fibroblast_EPIC | 0.284383907 | 2.93E-06 |
| Cancer associated fibroblast_MCPCOUNTER | 0.273044238 | 7.34E-06 |
| Cancer associated fibroblast_XCELL | 0.256004885 | 2.55E-05 |
| Common lymphoid progenitor_XCELL | -0.202134247 | 0.000957016 |
| Endothelial cell_EPIC | 0.160133439 | 0.009212513 |
| Endothelial cell_MCPCOUNTER | 0.195605528 | 0.001428814 |
| Hematopoietic stem cell_XCELL | 0.197818413 | 0.001233995 |
| Macrophage M1_CIBERSORT | -0.189045692 | 0.002036085 |
| Macrophage M1_QUANTISEQ | 0.154011995 | 0.012228165 |
| Macrophage/Monocyte_MCPCOUNTER | 0.171622459 | 0.005222656 |
| Macrophage_TIMER | 0.284828759 | 2.55E-06 |
| Mast cell_XCELL | 0.170018829 | 0.005613158 |
| Monocyte_MCPCOUNTER | 0.171622459 | 0.005222656 |
| Neutrophil_MCPCOUNTER | 0.355883753 | 3.44E-09 |
| Plasmacytoid dendritic cell_XCELL | -0.128411682 | 0.037052721 |
| stroma score_XCELL | 0.262573066 | 1.55E-05 |
| T cell CD4+ memory_XCELL | -0.139161234 | 0.023735275 |
| T cell CD4+ Th1_XCELL | -0.140767223 | 0.022152292 |
| T cell CD4+ Th2_XCELL | -0.242371138 | 6.92E-05 |
| T cell CD8+ naive_XCELL | -0.141531739 | 0.021431366 |
| T cell CD8+_EPIC | -0.17152463 | 0.005248713 |
| T cell CD8+_TIMER | 0.174580506 | 0.004440892 |
| T cell follicular helper_CIBERSORT | -0.203067266 | 0.000905225 |
| T cell follicular helper_CIBERSORT-ABS | -0.128743957 | 0.036561823 |
| T cell NK_XCELL | 0.141793208 | 0.021189511 |
| T cell regulatory (Tregs)_XCELL | 0.147826953 | 0.016228883 |
| uncharacterized cell_EPIC | -0.303495099 | 5.68E-07 |
| uncharacterized cell_QUANTISEQ | -0.133546818 | 0.030125324 |
